# Detection and quantification of dengue virus using a novel biosensor system based on dengue NS3 protease activity

**DOI:** 10.1371/journal.pone.0188170

**Published:** 2017-11-21

**Authors:** Ming-Shu Hsieh, Mei-Yu Chen, Chun-Hsiang Hsieh, Chien-Hsiung Pan, Guann-Yi Yu, Hsin-Wei Chen

**Affiliations:** 1 Graduate Institute of Life Sciences, National Defense Medical Center, Taipei, Taiwan, ROC; 2 National Institute of Infectious Diseases and Vaccinology, National Health Research Institutes, Zhunan, Miaoli, Taiwan, ROC; 3 Graduate Institute of Biomedical Sciences, China Medical University, Taichung, Taiwan, ROC; Fundaçao Oswaldo Cruz, BRAZIL

## Abstract

**Background:**

The traditional methods, plaque assays and immuno-focus assays, used to titrate infectious dengue virus (DENV) particles are time consuming and labor intensive. Here, we developed a DENV protease activity detection system (DENPADS) to visualize DENV infection in cells based on dengue protease activity.

**Methodology/Principal findings:**

Dengue NS3 protease cleaves NS4B-NS5. BHK-21 cells stably expressing the sensor module comprising DENV-2 NS4 and the 10 amino-terminal amino acids of NS5 (_N10_NS5) fused with the SV40 nuclear localization signal (NLS) and Cre recombinase (Cre), were generated. Cre is constrained outside the nucleus in the absence of NS3 activity but translocates into the nucleus through NS4B-NS5 cleavage when cells are infected with DENV. Nuclear translocation of Cre can trigger the reporter system, which contains a cis-loxP-flanked mCherry with three continuous stop codons following an SV40 polyA tail cDNA upstream of EGFP or mHRP cDNA. Our results show that DENPADS is an efficient and accurate method to titrate 4 DENV serotypes in 24 hours. Compared with current virus titration methods, the entire process is easy to perform, and the data are easily acquired.

**Conclusions/Significance:**

In this study, we demonstrate that DENPADS can be used to detect dengue viral infection through a fluorescence switch or HRP activity in the infected cells. This approach is sensitive with less incubation time and labor input. In addition, DENPADS can simultaneously evaluate the efficacy and cytotoxicity of potential anti-DENV candidates. Overall, DENPADS is a useful tool for dengue research.

## Introduction

Dengue virus (DENV) comprises 4 serotypes and has caused serious public health problems on a global scale [1]. According to a recent estimate, there are 390 million dengue infections annually [2]. Titration of viruses is an inevitable procedure for virology laboratories and is especially critical for clinical studies, vaccine development and anti-virus drug screening. Thus, an efficient and convenient approach for quantifying infectious virus will facilitate vaccine and drug development.

The “gold standard” method for titrating DENV is a plaque assay, which is based on plaque formation in an inoculated cell monolayer after dengue viral infection. Obtaining titration results from a standard plaque assay takes a long time (3 to >12 days) [3–6]. Another endpoint dilution assay is based on observing the cytopathic effect (CPE) after infection. The results are usually expressed as the median tissue culture infective dose (TCID_50_) [7, 8]. However, some clinical strains cannot be quantified by the plaque assay or TCID_50_ because of the absence of plaque formation or CPEs after infection [9]. An improved method, the immuno-fluorescent focus assay, detects virus based on immunostaining with dengue antigen [10]. This approach can shorten the incubation time to within 2 to 3 days, but extra steps for immunostaining are necessary, and the fluorescent focus must be observed and evaluated with a microscope. We used a chromogenic enzyme substrate method to yield visible colored foci, but the optimal incubation conditions required 3 to 4 days [11, 12]. Moreover, counting plaques or foci or observing CPEs is tedious and labor-intensive. Therefore, titrating a large number of samples using the above methods is challenging and time consuming. Recently, ELISA-based methods have been developed for DENV titration [13, 14]. These methods have the advantage of automated processes for reading and recording data and hence can be used to run a large number of titration tests. However, ELISA-based methods still require several days of incubation before virus can be quantified in cell culture. A quantitative reverse transcription PCR (qRT-PCR) approach is an accurate and fast method that allows several tests to be run. This approach is widely used to detect and quantify nucleic acids [15–17]. However, qRT-PCR results determine gene copy numbers, which do not completely represent the infective particle number, and accurate titers of infectious virus particles are not guaranteed. Other methods, such as flow cytometry, have also been used to measure DENV titers [18]. These methods are also antibody-dependent, and hence, they still require complicated immunostaining processes and may result in high cost.

The DENV genome encodes the structural proteins capsid (C), membrane precursor (prM), and envelope and the nonstructural proteins NS1, NS2, NS3, NS4, and NS5. These proteins initially form a precursor polyprotein that crosses through the endoplasmic reticulum (ER) membrane [19–21]. Cellular proteases and the virus-specific protease are responsible for cleaving the precursor polyprotein into functional proteins. The DENV protease consists of the amino-terminal domain of the NS3 protein and requires NS2B, a 14-kDa protein, as a co-factor to form a stable complex [22–25]. This heterodimeric protease cleaves specific sites at the junction of capsid-prM, NS2A-NS2B, NS2B-NS3, NS3-NS4A, and NS4B-NS5 [19, 26–28]. Using this characteristic cleavage capability, we can detect DENV-infected cells via protease activity. Medin et al. developed a DENV reporter plasmid, which contained an SV40 nuclear localization sequence and GFP downstream of the dengue viral cleavage site between NS4B and NS5 [29]. The dengue NS3 protease cleaved the GFP from the NS4B-NS5 junction, and the GFP was localized to the nucleus when cells were infected by DENV. Although viral infection can be determined by discrimination of GFP location under a fluorescence microscope, this approach is inconvenient for quantification of virus titers and makes it difficult to handle many samples at the same time.

In this study, we employed DENV NS3 protease activity to trigger a Cre-mediated reporter system. We show that this DENV protease activity detection system (DENPADS) is superior to traditional methods for determining dengue virus titers. DENPADS is not only efficient for titrating 4 infective DENV serotypes but can also be applied to high-throughput screening for drug discovery.

## Materials and methods

### Cells and viruses

Baby hamster kidney (BHK-21) cells (Bioresource Collection and Research Center, Hsinchu, Taiwan) were maintained in Dulbecco’s modified Eagle’s medium (DMEM) (Hyclone, Logan, UT, USA) supplemented with 10% fetal bovine serum (Biological Industries, Cromwell, CT, USA), 2 mM L-glutamine, 1.5 g/l sodium bicarbonate, 100 U/ml penicillin and 100 μg/ml streptomycin (Gibco, Thermo Fisher Scientific, Gran Island, NY, USA). DENPADS cells (developed in this study and derived from BHK-21) were maintained in the same medium additionally containing 400 μg/ml Zeocin^™^ (Invitrogen, Thermo Fisher Scientific, Waltham, MA, USA) and 800 μg/ml Geneticin (Gibco). Cells were incubated at 37°C in a humidified chamber with 5% CO_2_.

DENV-1/Hawaii, DENV-2/16681, DENV-3/H-87 and DENV-4/H241 were used for this study. All the viruses were propagated in Vero cells, and most viral titers were determined using focus-forming assays with BHK-21 cells [12]. The Zika virus/PRVABC59 titers were determined using a plaque assay. The Japanese encephalitis virus (JEV)/Beijing-1 was amplified and purified as previously described [30].

### Plasmid construction

The NS4B/_N10_NS5 cDNA (GenBank: M84727.1, 6826–7599) was obtained from DENV-2 16681 virus RNA via reverse transcription and then amplified; 5’-XhoI and 3’-AflII-EcoRI restriction sites were generated using PCR. The PCR NS4B/_N10_NS5 fragment was cloned into pcDNA3.1(-) using XhoI/AflII restriction endonucleases to create the plasmid pNS4B/_N10_NS5. The Cre recombinase cDNA sequence (GenBank: AB449974.1, 114–1142) was amplified via PCR from CD4cre mouse (Jackson Laboratory, Bar Harbor, ME, USA) genomic DNA [31]. The SV40 NLS (ccaagaagaagaggaaggtg, encoding PKKKRKVG) was fused to the 5’ end of the Cre recombinase cDNA sequence using PCR to generate 5’-EcoRI and 3’-AflII-HindIII restriction sites. Then, the PCR NLS-Cre fragment was cloned into pNS4B/_N10_NS5 using EcoRI/AflII endonucleases to establish the plasmid pSen-Cre. To establish the reporter module pRep-enhanced green fluorescent protein (EGFP) or pRep-membrane-targeted horseradish peroxidase (mHRP), the DNA fragment “AflII-loxp-mcherry-stop codon-SV40 polyA terminator-loxp-BamHI” (oligo synthesized by Genomics Inc., New Taipei, Taiwan) was first cloned into pcDNA4/TO/myc-HisA using AflII/BamHI endonucleases to create the plasmid pLoxp-flanked-mcherry. Next, the fragment “BamHI-EGFP-XbaI” or “BamHI-mHRP-XbaI” was obtained via PCR and oligo synthesis (Genomics Inc.), respectively. Then, each fragment was cloned into the plasmid pLoxp-flanked-mcherry using BamHI/XbaI endonucleases to construct pRep-EGFP or pRep-mHRP. The NS2B/NS3 cDNA (GenBank: M84727.1, 4132–6375) was obtained from DENV-2 16681 virus RNA via reverse transcription and then amplified by PCR. To establish the plasmid pCre and pNS2B3, the Cre recombinase and NS2B/NS3 cDNA sequence was amplified by PCR and generate 5’-XhoI-NotI and 3’-AflII-HindIII restriction sites. Then, the PCR Cre and NS2B/NS33 fragment was cloned into pcDNA3.1(-) using XhoI/AflII endonucleases to create pCre and pNS2B3. All primers used for PCR are shown in S1 Table.

### Transfection

BHK-21 cells were seeded in 24-well plates at 7×10^4^ cells per well. For some experiments, cells were seeded in 24-well plates with a glass coverslip bottom. After 24 hours of incubation, the culture medium was replaced with 0.5 ml per well of fresh culture medium 30 minutes before transfection. For each well, 1.5 μl of PolyJet^™^ Reagent (SignaGen Laboratories, Rockville, MD, USA) was diluted in 25 μl of serum-free medium, and 0.5 μg of plasmid was diluted in 25 μl of serum-free medium. Then, the PolyJet^™^ solution was added to the plasmid solution, and the mixture was vortexed briefly followed by incubation for 15 min at room temperature. The PolyJet^™^/DNA complex was then added to the cells. After 12 hours of transfection, PolyJet^™^/DNA complex-containing medium was removed and replaced with fresh complete medium.

### Confocal imaging

To analyze the localization of target protein expression, BHK-21 cells were seeded in 24-well plates with a glass coverslip bottom at 7×10^4^ cells per well. After 24 hours of incubation, the cells were transfected with pCre, pNS2B3 or pSen-Cre. At 24 hours post-transfection, the cells were fixed with 3.7% paraformaldehyde in PBS for 30 min and permeabilized with 0.5% TritonX-100 for 10 min. Cells were then blocked with 1% BSA in PBS followed by incubation overnight with primary anti-Cre Recombinase antibody (Cell Signaling Technology, Danvers, MA, USA), anti-Flavivirus NS3 antibody (Yao-Hong Biotechnology, Taipei, Taiwan), or anti-dengue NS4B antibody (GeneTex, San Antonio, TX, USA). After incubation with primary antibodies, the cells were washed three times with PBS and incubated with Alexa Fluor-488-conjugated secondary antibody or Alexa Fluor-594-conjugated secondary antibody (Invitrogen) for 1 hour at room temperature. Cell nuclei were counterstained with Hoechst 33342 (Invitrogen). The coverslips were affixed onto glass slides and imaged using Leica TCS SP5II confocal microscope (Leica Microsystems, Wetzlar, Germany).

### Selection of monoclonal cell lines

**Fig 1:**
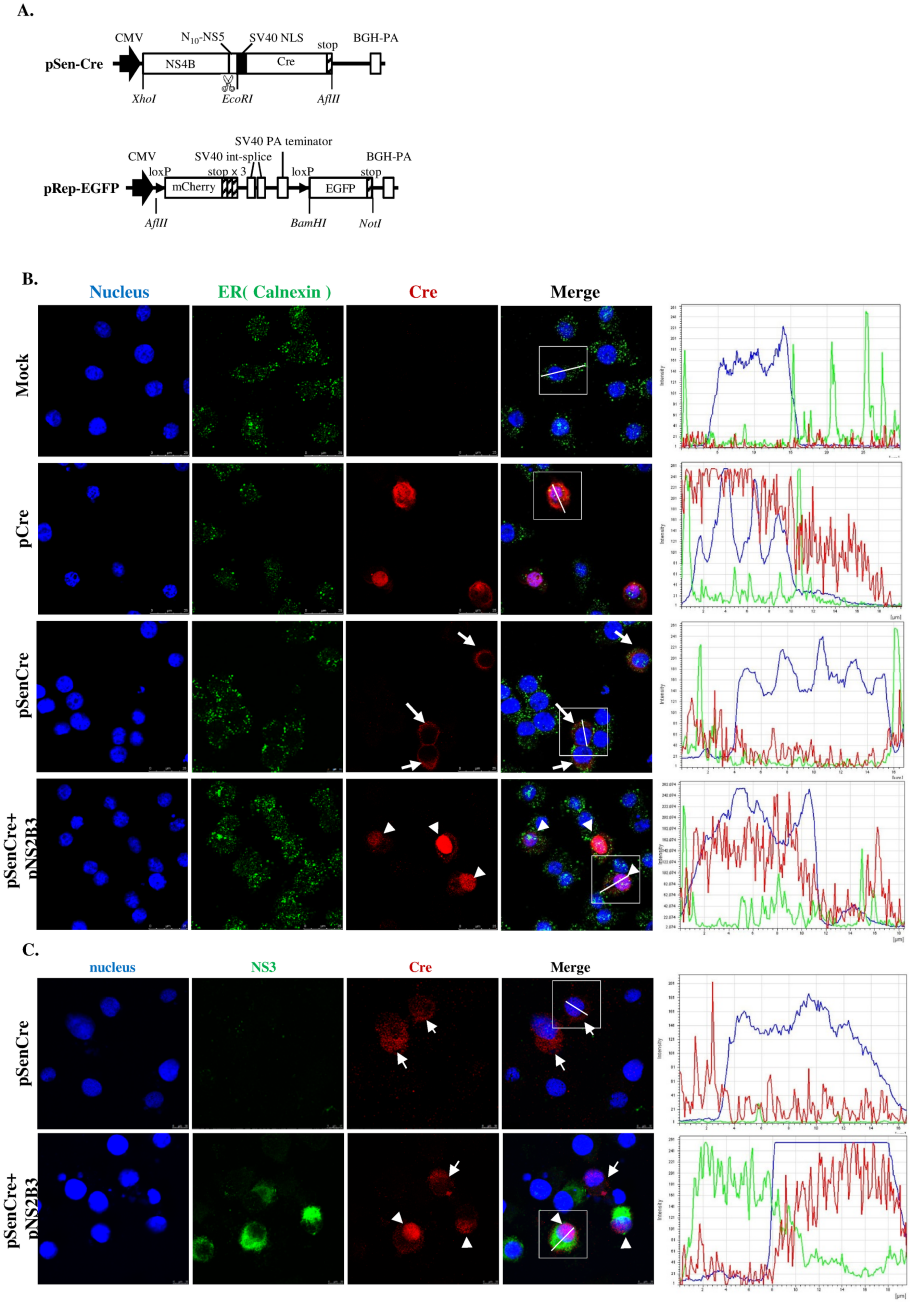
Construction of DENPADS plasmids and characterization of the sensor module. (A) pSen-Cre and pRep-EGFP were constructed as the sensor and reporter modules, respectively. The cleavage site of the DENV NS2B3 protease is indicated. BHK-21 cells were transfected with pSen-Cre in the presence or absence of pNS2B3. Localization of Cre (red) and (B) ER membranes (calnexin, green) or (C) NS3 (green) were detected by immunostaining. Nuclei were counterstained with Hoechst (blue). The fluorescence intensity was analyzed by LEICA Application Suite software (magnification, 100×). Cells transfected with pCre served as controls.

To establish Rep-EGFP or Rep-mHRP stable clone cell lines, BHK-21 cells were transfected with pRep-EGFP or pRep-mHRP. After 24 hours of transfection, the cells were seeded into 96-well plates at limiting dilution and incubated in culture medium containing 400 μg/ml Zeocin^™^ (Invitrogen). Then, the plates were incubated at 37°C in a humidified CO_2_ incubator until a single colony could be identified in each well. Then, the colonies were subcultured from the wells into larger vessels. The Rep-EGFP or Rep-mHRP monoclonal cell lines were verified by selection of the EGFP^+^ or HRP^+^ cells after pCre transfection. To establish F-DENPADS and H-DENPADS cell lines, pSen-Cre (Fig 1) was transfected into Rep-EGFP or Rep-mHRP stable cell lines, and cells were cultured at limiting dilution as described above. Then, the cells were incubated in culture medium containing 400 μg/ml Zeocin^™^ and 800 μg/ml Geneticin (Gibco). F-DENPADS cell lines and H-DENPADS cell lines were verified by selection of the EGFP^+^ or HRP^+^ cells after DENV-2 infection, and the expression of Sen-Cre was verified by western blot.

### Western blotting

Transfection or infection of the Rep-EGFP cell line (pRep-EGFP^+^) or F-DENPADS cell line (pRep-EGFP^+^ and pSen-Cre^+^) was performed as described above. Cell lysates were collected after incubation of cells with RIPA lysis buffer (Thermo Scientific, Thermo Fisher Scientific, Waltham, MA, USA) supplemented with Halt^™^ Protease Inhibitor Cocktail (Thermo Scientific). The total lysate protein was quantified using a Pierce^™^ BCA Protein Assay Kit (Thermo Scientific). Equal amounts of protein were run on 10 or 12% sodium dodecyl sulfate (SDS)-polyacrylamide gels after addition of equal volumes of 4× Laemmli loading buffer (65.5 mM Tris-HCl, pH 6.8, 2% SDS, 26.3% glycerol, and 0.01% bromophenol blue). Each sample was loaded in an SDS-polyacrylamide gel and electrophoretically separated using common methods. The proteins were transferred to nitrocellulose membranes, and the resulting blots were blocked with 5% non-fat dry milk in PBST for 1 hour at room temperature. Then, the blots were incubated with anti-Cre Recombinase (Cell Signaling Technology) and anti-Actin (GeneTex) antibodies on a shaker overnight at 4°C. After three washes with PBST, the blots were incubated with HRP-conjugated goat anti-rabbit antibody (GeneTex) for 1 hour at room temperature following three washes. The proteins were finally detected via chemiluminescence (SuperSignal^™^ West Pico Chemiluminescent Substrate, Thermo Scientific) and exposed to autoradiographic film.

### Quantification of virus titer using F-DENPADS

F-DENPADS cells were seeded prior to infection at a density of 7×10^4^ cells per well in 24-well plates. After 24 hours of incubation, the culture medium was replaced with 180 μl of serum-free DMEM, and cells were infected with 50 μl of a 10-fold serial dilution of standard DENV-2 or DENV-2 samples for 3 hours. Then, the serum-free medium was replaced with DMEM containing 2% FBS. Cells were collected at 24, 48, and 72 hours post-infection and fixed with IC Fixation Buffer (eBioscience, Thermo Fisher Scientific, San Diego, CA, USA) for 5 min. The cells were washed one time and resuspended in PBS. Then, cells were analyzed on a FACS flow cytometer (BD FACSCalibur^™^) to detect EGFP-positive cells. At least 30,000 events were collected for each sample. To prepare a standard curve, the average of the percentage of EGFP-positive cells versus the virus titer measured from the immuno-focus assay were plotted in terms of FFU/well or FFU/ml. The virus titer of each sample was determined by interpolating from the standard curve.

### Quantification of virus titer using H-DENPADS

H-DENPADS cells were seeded prior to infection at a density of 1.7×10^4^ cells per well in 96-well plates. After 24 hours of incubation, the culture medium was replaced with 50 μl of serum-free DMEM, and cells were infected with 50 μl of a 10-fold serial dilution of standard DENV or other virus type samples for 3 hours. Then, the serum-free medium was replaced with DMEM containing 2% FBS, and cells were incubated for 21 hours. The cells were collected and fixed with IC Fixation Buffer (eBioscience) for 5 min. Following two washes with PBS, the 3,3',5,5'-tetramethylbenzidine (TMB) soluble substrate (1-Step^™^ Ultra TMB-ELISA Substrate Solution, Thermo Scientific) was added to each well, and cells were incubated for 10 min at room temperature. After the reaction, the supernatant was placed into an ELISA plate, and the reaction was stopped by adding 2 N H_2_SO_4_. The OD450 nm value was estimated with a spectrophotometer. To prepare a standard curve, the average blank-corrected OD450 nm value versus the virus titer measured from the immuno-focus assay were plotted in terms of FFU/well or FFU/ml. The virus titer of each sample was determined by interpolating from the standard curve.

### Antiviral compounds

The anti-virus compounds 2’-C-methyladenosine (2-CM) (Carbosynth, Compton, Berkshire, UK) and mycophenolic acid (MPA) (Sigma-Aldrich, St. Louis, MO, USA) were dissolved in DMSO unless otherwise stated to produce 20 mM stock solutions, and aliquots were stored at -20°C.

### Virus inhibition assay using H-DENPADS and virus yield reduction assays

H-DENPADS cells were seeded at a density of 1.7×10^4^ cells/well in 96 well plates and incubated for 24 hours. Cells were infected with DENV-2 at an m.o.i of 5 in serum-free medium for 3 hours. The serum-free medium was replaced with drug-containing (at concentrations of 2.5, 5, 10, 15, or 20 μM) or no drug-containing culture medium and incubated for an additional 21 hours. The degree of virus infection was determined by the OD450 nm value corrected by virus titer per well as described above. Simultaneously, cell culture supernatants were collected at 24 hours post-infection and either used immediately or stored at -80°C before being subjected to immuno-focus assays. The virus titers in supernatants were determined using immuno-focus assays.

### Cytotoxicity assay

The cytotoxic effect of anti-dengue drugs was determined by the number of viable cells in a proliferation assay using CellTiter 96^®^ AQueous One Solution Reagent (Promega, Madison, WI, USA) containing the tetrazolium compound 3-(4,5-dimethylthiazol-2-yl)-5-(3-carboxymethoxyphenyl)-2-(4-sulfophenyl)-2H-tetrazolium, inner salt (MTS). H-DENPADS cells were plated at a density of 1.7×10^4^ cells per well in 96-well plates containing 100 μl of culture medium. After 24 hours of incubation, the culture medium was replaced with serum-free medium for 3 hours. The serum-free medium was replaced with drug-containing (at concentrations of 2.5, 5, 10, 15, or 20 μM) or no drug-containing culture medium, and the cells were incubated for an additional 21 hours. MTS reagent was then added to each well and incubated with cells for 2 hours at 37°C in a humidified 5% CO_2_ atmosphere. Then, the OD490 nm value was estimated with a spectrophotometer.

## Results

### Construction and characterization of DENPADS

In this study, we employed dengue viral protease for detection of DENV. Two plasmids, pSen-Cre and pRep-EGFP, were constructed for proof-of-concept. In the sensor plasmid construct (pSen-Cre), the DENV-2 NS4 sequence and 10 amino-terminal amino acids of NS5 (_N10_NS5) were linked with the SV40 nuclear localization signal (NLS) and Cre recombinase (Cre). In addition, cDNA encoding loxP-flanked mCherry with 3 stop codons upstream of EGFP cDNA was cloned into a pcDNA4 vector to construct the reporter plasmid, pRep-EGFP. The structure of the pSen-Cre and pRep-EGFP constructs and the relative positions of the genes are shown in Fig 1A. To test the function of the sensor plasmid, we first transfected BHK-21 cells with pSen-Cre and examined the Cre localization in the presence or absence of the dengue viral protease (NS3) and its co-factor (NS2B). As shown in Fig 1B, the majority of Cre was not localized in the nucleus without co-transfection with pNS2B3, which encoded the dengue NS3 protease and the co-factor NS2B. After co-transfection of BHK-21 cells with pSen-Cre and pNS2B3, Cre was guided into the nucleus. We further confirmed that Cre translocation to the nucleus was only observed in NS3-expressing cells but not in NS3-negative cells (Fig 1C). However, NS4B remained outside of the nucleus regardless of whether cells were co-transfected with NS2B/3 (S1 Fig). These results suggest that Cre was normally localized in the ER through fusion to the C terminus of NS4B-_N10_NS5 until it was freed by the viral protease NS2B/NS3.

**Fig 2:**
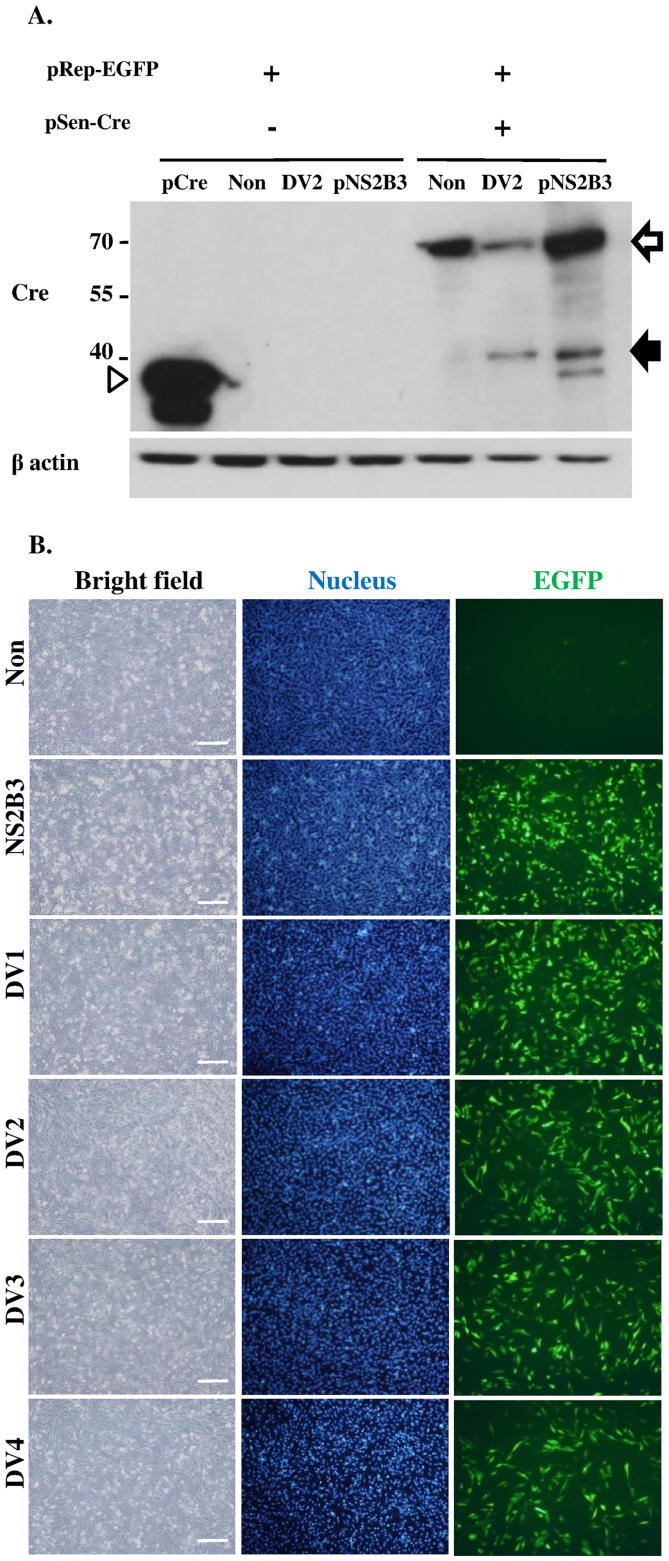
Characterization of DENPADS. (A) The pRep-EGFP stable clone or F-DENPADS cells were transfected with pNS2B3 or infected with DENV-2 (m.o.i. = 5). Cells transfected with pCre or mock infected cells served as controls. Cell lysates were harvested and then analyzed by immunoblotting using anti-Cre and anti-β-actin antibodies 48 hours after transfection or infection. The cleavage product (solid arrow) represents the fragment cleaved from the NS4B-_N10_NS5/NLS-Cre fusion protein (hollow arrow) by NS2B3 at the region between NS4B and NS5 during DENV infection or NS3 presentation. The hollow arrowhead represents the Cre protein (~38 kDa), which is encoded by pCre. (B) F-DENPADS cells were infected with 4 DENV serotypes at an m.o.i. of 5. Cells were fixed and analyzed 48 hours post-infection using fluorescence microscopy. Scale bar, 250 μm.

To further verify that Cre translocation was induced by proteolytic processing of the cleavage site between NS4B and NS5, subsequently triggering the reporter response, we established DENPADS in BHK-21 cells. Cells were sequentially transfected with the pRep-EGFP and pSen-Cre plasmids to generate the fluorescence-based DENPADS (F-DENPADS) stable cell line. F-DENPADS cells were infected with DENV-2 at an m.o.i. of 5 or transfected for 48 hours with pNS2B3 to express viral protease. Immunoblotting analysis was performed on cell lysates using an antibody against Cre. Uninfected F-DENPADS cells showed a single protein band of approximately 70 kDa, which is the expected size of the NS4B-_N10_NS5/NLS-Cre fusion protein (Fig 2A hollow arrow). When cells were infected with DENV-2 or transfected with pNS2B3, an additional smaller protein band of ~40 kDa (Fig 2A solid arrow) was revealed. The size of the small protein band was consistent with the expected size of the cleavage fragment _N10_NS5/NLS-Cre, which was slightly larger than the Cre control (~38 kDa) (Fig 2A hollow arrowhead). These results suggest that NS4B-_N10_NS5/NLS-Cre was cleaved during DENV 2 infection, releasing the Cre-containing fragment, which translocated into the nucleus.

To further examine whether F-DENPADS could trigger EGFP expression by DENV infection, F-DENPADS cells were transfected with pNS2B3 or infected with DENV. Uninfected cells only showed punctate background level EGFP expression. In contrast, pNS2B3-transfected or DENV-infected cultures displayed distinct EGFP expression (Fig 2B). The results show that F-DENPADS can detect the infection of 4 dengue serotypes.

### Quantification of DENV titer by F-DENPADS

**Fig 3:**
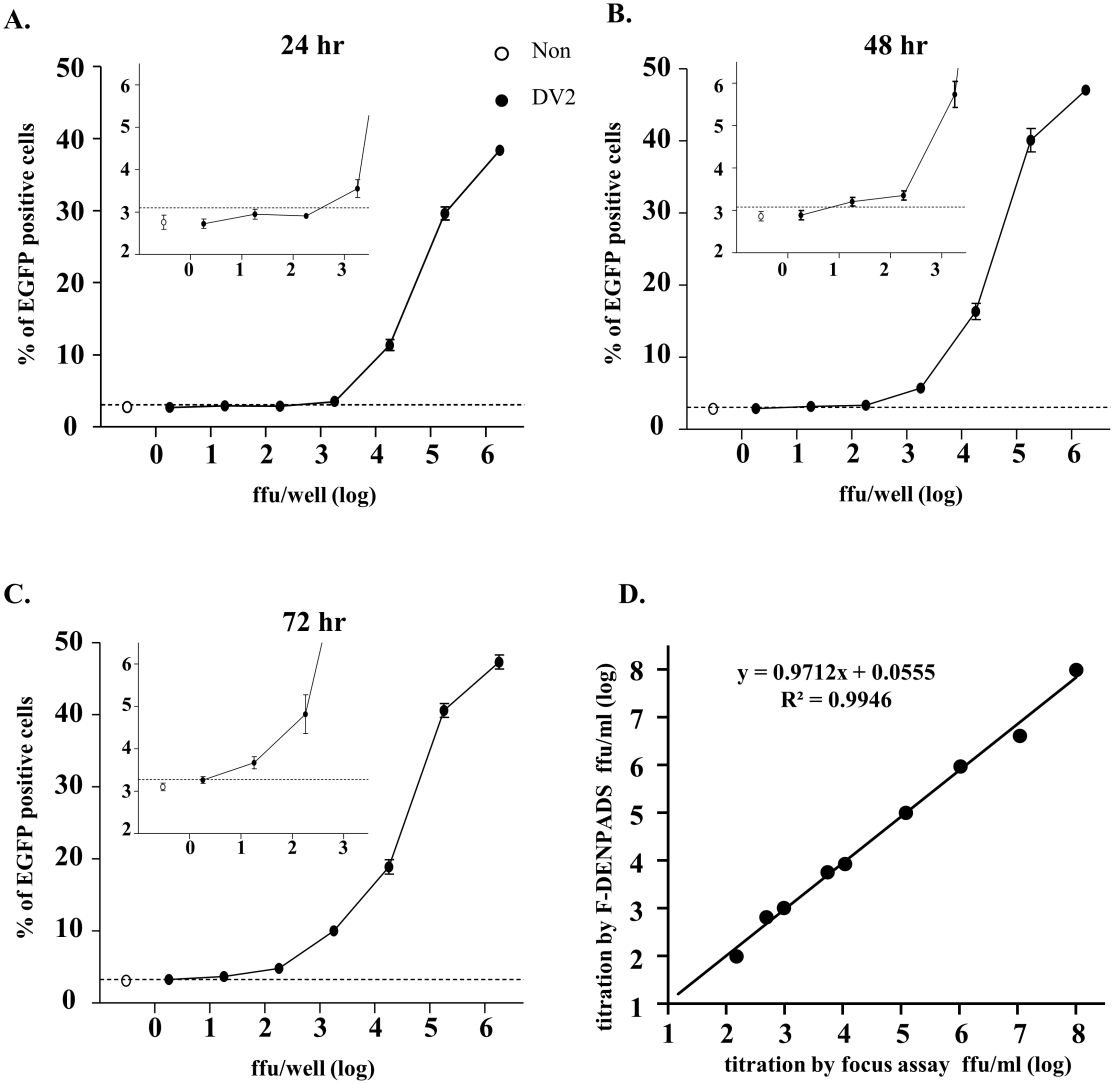
Determination of DENV titers by F-DENPADS. F-DENPADS cells were seeded in 24-well plates. After incubation for 24 hours, the cells were infected with a 10-fold serial dilution of DENV-2. EGFP expression was examined via flow cytometry 24 (A), 48 (B), and 72 (C) hours after infection. The percentage of EGFP-positive cells versus virus titers is plotted. The hollow dot represents the percentage of EGFP-positive cells in the non-infection group and is defined as the background value. Partially enlarged views between log 0 to 3 FFU/well are presented in the upper-left within the diagram. The data shown are the mean values ± standard deviation obtained from 3 independent experiments. The dotted lines indicate the mean plus a 2-fold standard deviation of the non-infection group. (D) Virus titers of 9 DENV samples were converted by the standard curve that was obtained from F-DENPADS cells incubated for 72 hours (in Fig 3C). Virus titers were determined in parallel by an immuno-focus assay. The correlation between the two methods is shown.

In view of our finding that dengue viral infection could be detected in F-DENPADS cells, we next evaluated the F-DENPADS cells for DENV quantification. The F-DENPADS cells were infected with a 10-fold serial dilution of DENV-2 (starting at 1.8×10^6^ focus-forming units (FFU)/well), and then, EGFP-expressing cells were monitored 24 (Fig 3A), 48 (Fig 3B), and 72 (Fig 3C) hours after DENV-2 infection. The percentage of EGFP^+^ cells correlated with the virus titers for all the time points examined. When the percentage of EGFP^+^ cells was above the mean of the non-infection control plus a 2-fold standard deviation, indicated by the dotted line, the cells were considered positive for viral infection. The detection limits (defined as non-infection control plus a 2-fold standard deviation), estimated from the titration curve by interpolation, were 645, 8.5, and 1.9 FFU/well for incubation for 24, 48, and 72 hours, respectively. These results indicate that the sensitivity of F-DENPADS increases as incubation times are extended.

To validate F-DENPADS for DENV quantitation, 9 samples with various virus titers were tested. The virus titers of the 9 samples were determined in parallel with immuno-focus assays. As shown in Fig 3D, the correlation analysis performed between titers obtained using F-DENPADS and those obtained using the immuno-focus assay revealed a high Pearson correlation coefficient (R) of 0.9972 (R^2^ = 0.9946). These results indicate that F-DENPADS has high accuracy and reliability.

### The activity of F-DENPADS in detection of other flavivirus infections

**Fig 4:**
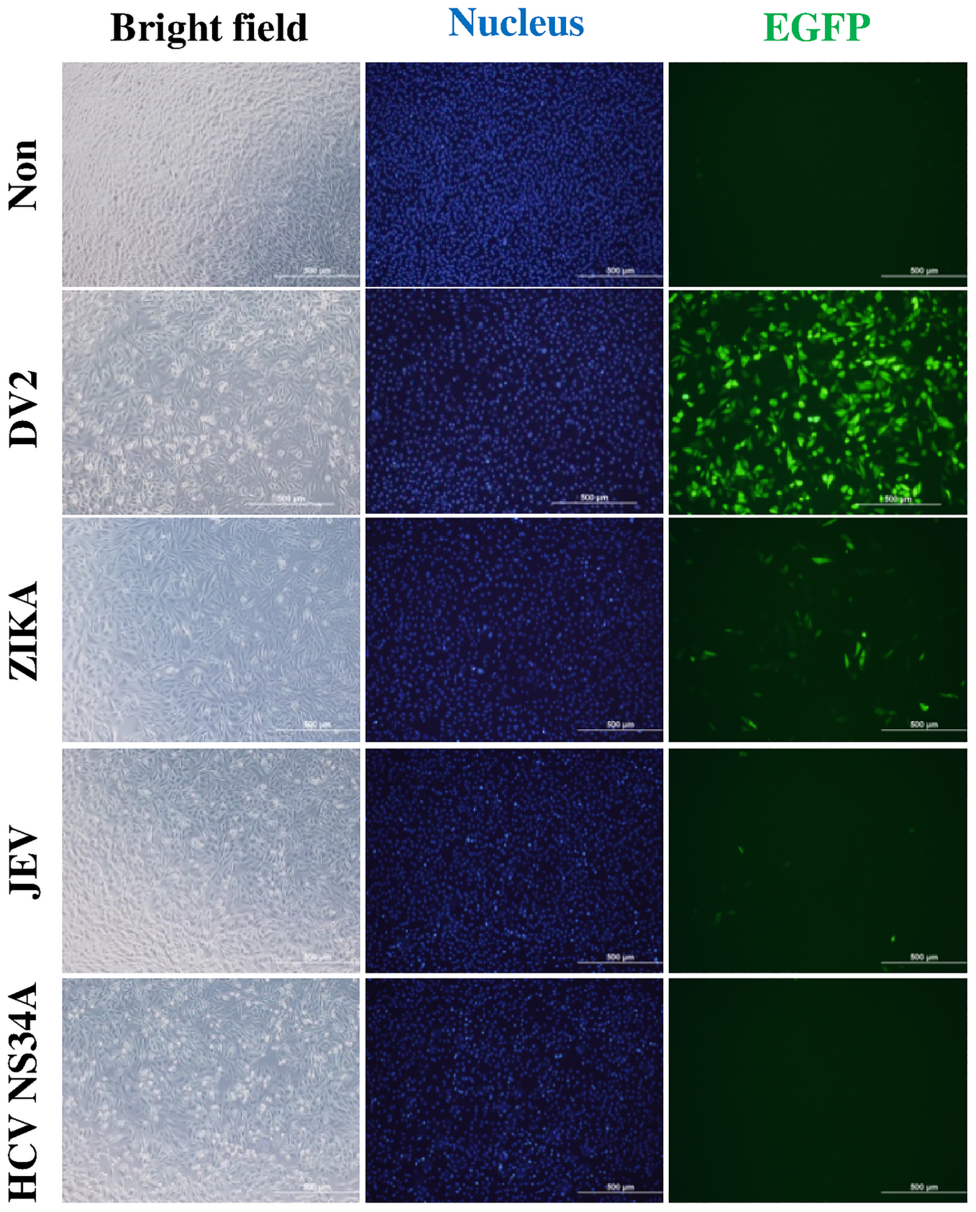
Efficiency of F-DENPADS in detection of other flaviviruses. F-DENPADS cells were infected with DENV-2, Zika virus, or JEV at an m.o.i. of 5 or transfected with pHCV NS3/4A-Flag. Cells were examined for EGFP expression 48 hours post-infection using fluorescence microscopy. Scale bar, 500 μm.

To evaluate whether F-DENPADS could be applied to detect other flaviviruses, F-DENPADS cells were infected with DENV-2, Zika virus, or JEV at an m.o.i. of 5 or transfected with pHCV-Flag-NS3/4A, which encoded Hepatitis C virus (HCV) protease NS3/4A with a Flag tag. After incubation for 48 hours, Zika-infected cultures displayed a distinct EGFP signal, but the expression of EGFP was still conspicuously lower than that observed with DENV-2 infection. In contrast, JEV-infected or HCV NS3/4A transfected cells showed punctate fluorescence comparable to that observed in uninfected cells (Fig 4). In addition, virus infection and HCV NS3/4A expression were confirmed by immunostaining after the fluorescence detection (S2 Fig). These results indicate that F-DENPADS is not sensitive to other flaviviruses.

### Evaluation of a membrane-targeted HRP reporter system

**Fig 5:**
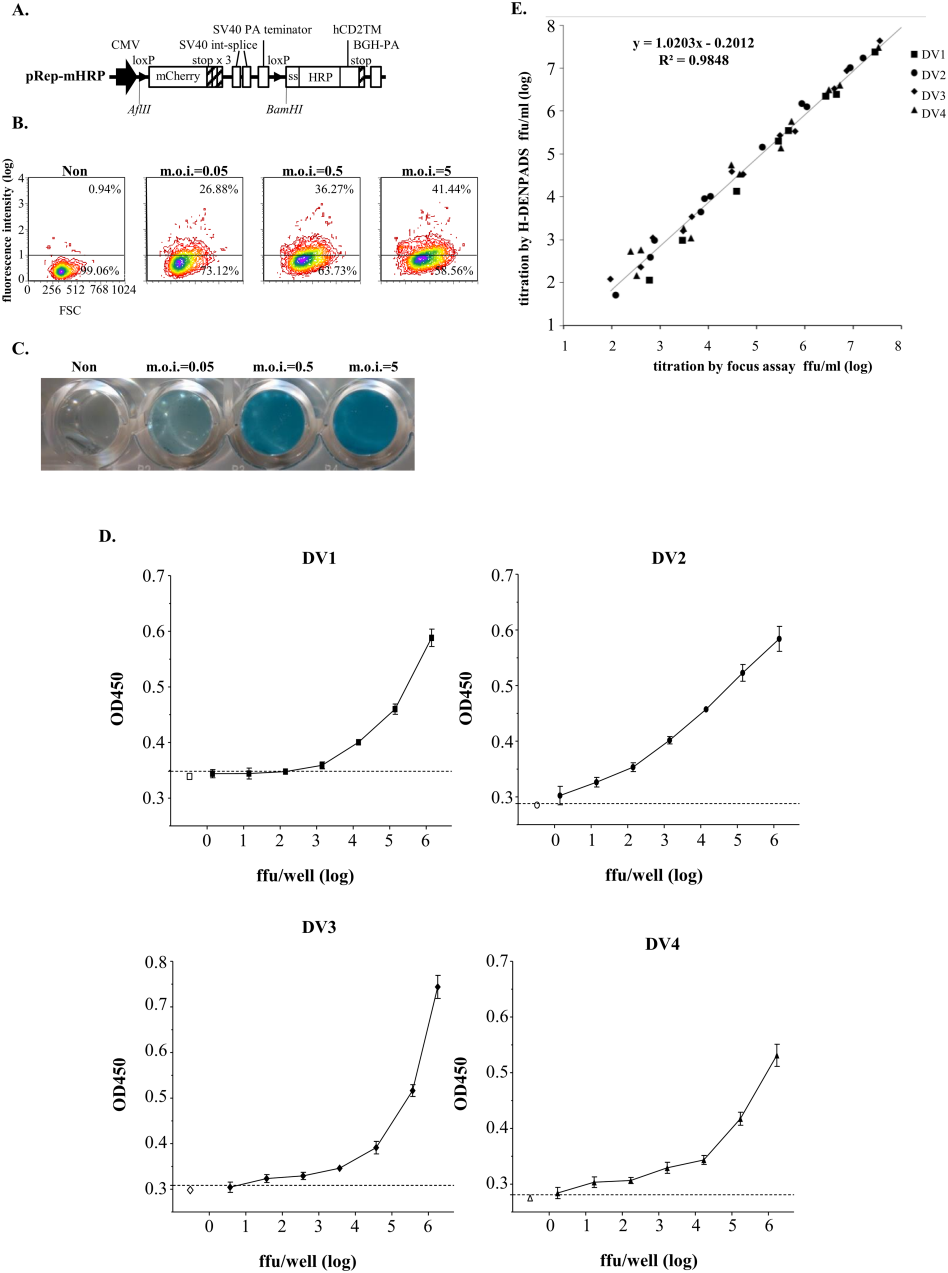
Determination of DENV titers by H-DENPADS. (A) pRep-mHRP was constructed as a membrane-targeted HRP reporter module. (B) H-DENPADS cells were infected with DENV-2 for 48 hours. The expression of mHRP was examined by surface staining with anti-HRP antibodies using flow cytometry. (C) The function of mHRP was evaluated by color development after adding TMB substrate. (D) H-DENPADS cells were seeded in 96-well plates. After incubation for 24 hours, cells were infected with a 10-fold serial dilution of DENV-1, -2, -3, or -4. TMB substrate was added 24 hours after infection. The OD450 nm was examined with a spectrophotometer. The data are representative of 3 different experiments performed in hexaplicate. The dotted lines indicate the mean plus a 2-fold standard deviation of the non-infection group. (E) Virus titers of 43 DENV samples were converted by the standard curve, which was obtained from Fig 5D. Virus titers were determined in parallel by an immuno-focus assay. The correlation between the two methods is shown.

To test another widely used reporter system, mHRP was substituted for EGFP in the pRep-EGFP plasmid. The mHRP was designed based on a previous study [32] and composed by adding an N-terminal signal sequence to the cDNA of HRP and placing the transmembrane domain from human CD2 in the C-terminus (Fig 5A). Cells were sequentially transfected with the reporter plasmid pRep-mHRP and pSen-Cre (Fig 1A) to generate a stable mHRP-based DENPADS (H-DENPADS) cell line. To validate H-DENPADS, the cells were infected with DENV-2 at 0.05, 0.5, and 5 m.o.i. After 48 hours of infection, the mHRP expression levels were determined by surface staining with anti-HRP antibody. Mock infected cells showed a background value of <1% HRP-positive cells. Infected cells showed an apparent increase in mHRP expression in a dose-dependent manner (Fig 5B). Importantly, mHRP was functional, as indicated by the blue color that developed when the TMB substrate was added (Fig 5C). These results indicate that functional mHRP was triggered by DENV infection.

Next, we further evaluated the ability of H-DENPADS to quantify DENV within a short incubation time (24 hours). H-DENPADS cells were infected with a 10-fold serial dilution of DENV (starting at 1.4×10^6^ FFU/well for DENV-1, 6.0×10^6^ FFU/well for DENV-2, 3.7×10^6^ FFU/well for DENV-3, and 1.7×10^6^ FFU/well for DENV-4), and then, substrate (TMB) was added for color development 24 hours after infection. The OD450 nm values associated with the virus titer were quantitated by immuno-focus assays (Fig 5D). When the OD450 nm value was above the mean of the non-infection control plus a 2-fold standard deviation, as indicated by the dotted line, the cells were considered positive for viral infection. The detection limits (defined as non-infection control plus a 2-fold standard deviation) for DENV-1, -2, -3 and -4, estimated from the titration curve by interpolation, were 159, 1.8, 6 and 1.8 FFU/well, respectively.

To validate H-DENPADS for DENV quantitation, 43 DENV samples with various virus titers were quantitated by H-DENPADS. The virus titers of the 43 samples were also determined in parallel by immuno-focus assays. As shown in Fig 5E, the correlation analysis performed between titers obtained using H-DENPADS and those obtained using immuno-focus assays revealed a high correlation coefficient (R) of 0.9923 (R^2^ = 0.9848). These results indicate that H-DENPADS can be used to determine titers of 4 DENV serotypes.

### Application of H-DENPADS in drug discovery

**Fig 6:**
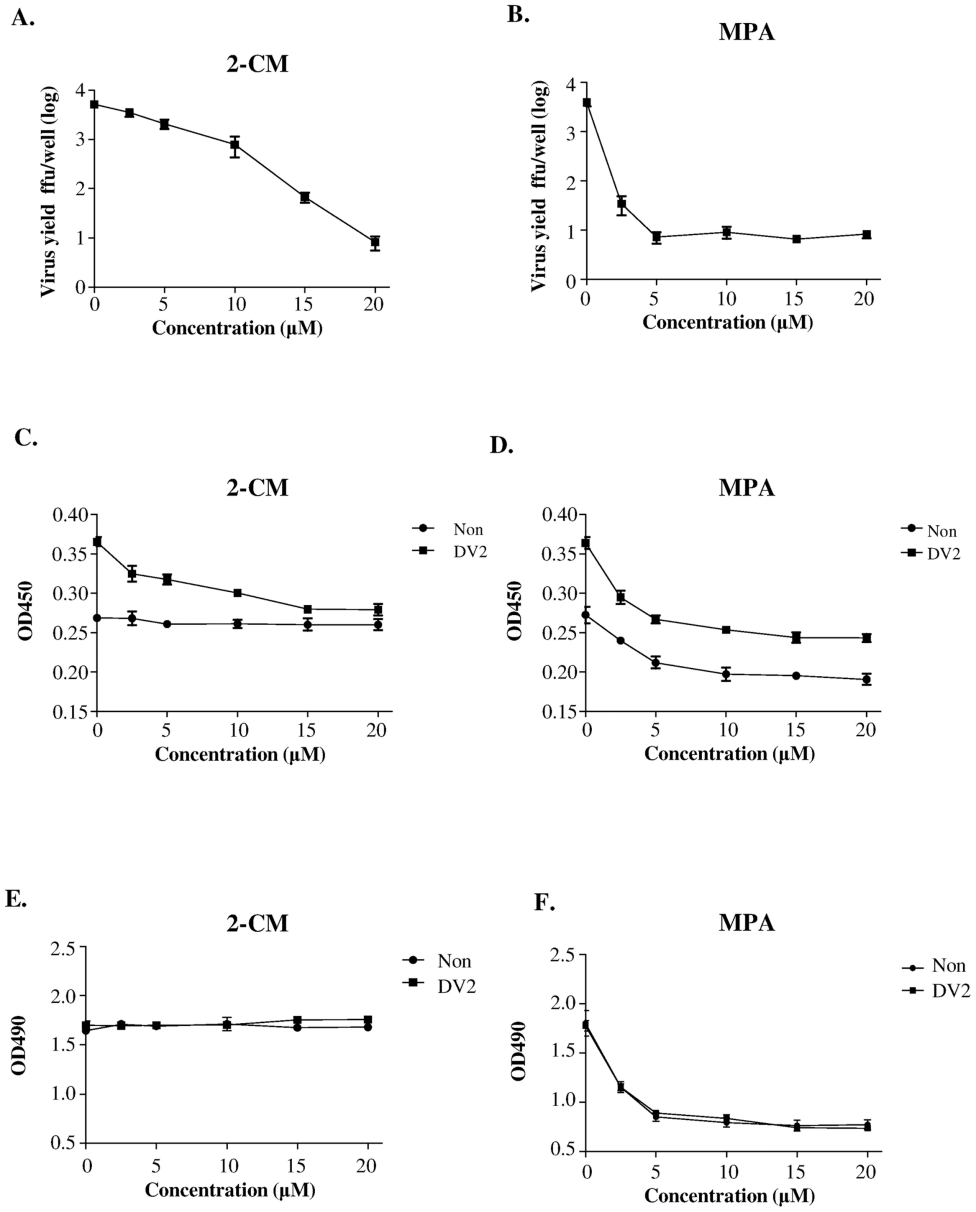
H-DENPADS as an anti-DENV drug screening platform. H-DENPADS cells were exposed to DENV-2 at an m.o.i. of 5 in serum-free medium for 3 hours. After infection, cells were cultured in culture medium with various concentrations of (A, C, E) 2’-C-methyladenosine (2-CM) or (B, D, F) mycophenolic acid (MPA) as indicated. Mock infected cells served as the controls. Virus titers in the culture medium after infection for 24 hours were determined by immuno-focus assays (A, B) or H-DENPADS (C, D). The cytotoxic effect was analyzed with MTS assays (E, F). The data are representative of 3 different experiments performed in hexaplicate.

To evaluate whether H-DENPADS is a powerful tool for identifying anti-virus drugs, two commonly used anti-DENV candidates, 2’-C-methyladenosine (2-CM) [33, 34] and mycophenolic acid (MPA) [34–37], were tested as representative drugs. The efficacy of the two candidates were first verified by virus yield reduction assays (Fig 6A and 6B). Simultaneously, the two candidates were tested by H-DENPADS, and virus titers were indicated by the OD450 nm values. As shown in Fig 6C and 6D, both 2-CM and MPA led to apparent decreases in OD450 nm values in a dose-dependent manner under DENV-2 infection. The results obtained from the virus yield reduction assays and H-DENPADS were consistent with each other. These results suggest that H-DENPADS could be used as a drug discovery platform.

We noticed that the OD450 nm values obtained after 2-CM treatment without infection were invariant, but the OD450 nm values after MPA treatment appeared to decrease as the concentration increased in the non-infection groups (Fig 6C and 6D). These results may be due to the strong cytotoxic effects of MPA [38, 39]. As shown in Figs 6E and S3A, addition of 2-CM did not significantly reduce cell numbers regardless of the presence or absence of infection. In contrast, the cell numbers were consistently decreased when MPA was added, with and without DENV infection (Figs 6F and S3B). Taken together, these results indicate that H-DENPADS can simultaneously evaluate the efficacy and cytotoxicity of potential anti-DENV candidates.

## Discussion

In the present study, a novel method, termed DENPADS, was developed to quantify infectious DENV. Our results show that DENPADS can easily detect or quantify DENV by determining EGFP expression (Fig 3A–3D) or HRP activity (Fig 5D and 5E). DENPADS is sensitive to 4 DENV serotypes (Fig 2B) but not to other flaviviruses that we tested (Fig 4). The DENV-2 NS4B-_N10_NS5 gene was utilized to construct the sensor module pSen-Cre (Fig 1A). The range within P2-P2’ of the NS3 protease cleavage site has been shown to be critical for efficient cleavage [40, 41]. The amino acid sequences around the NS4B-NS5 boundary are constant within the position P2-P3’ (K/R-R-G-T-G) among the 4 DENV serotypes (S4A Fig). The P2-P3’ positions of Zika virus and JEV are similar to DENV, except for P2’ of Zika virus and P2’-P3’ of JEV. Notably, the P2-P3’ positions of HCV are different from those of DENV. In addition, the homologies of the DENV-2 NS3 protease among other DENV serotypes are ≥70%. In contrast, the homologies of DENV-2 NS3 protease among the other flaviviruses we tested are ≤56% (S4B Fig). These findings may be part of reason that DENPADS is efficient in detecting the 4 DENV serotypes but not the other tested flaviviruses.

The plaque assay, which involves counting plaque-forming units, is the gold standard method for titrating DENV. The immuno-focus assay, an alternative to the standard plaque assay, is expressed as FFU through detection of viral antigen. Both methods require several days to form countable plaques or FFU [3–6, 42, 43]. Recently, a novel NS1 antigen capture ELISA-based method was developed for titration of DENV [13]. A 6-day incubation appeared to be sufficient for the infected cells to produce enough NS1 protein to be detected by ELISA [13]. We show that the incubation time of DENPADS is less than 3 days (Figs 3A–3C and 5D). Notably, H-DENPADS only takes 24 hours. The detection limits of H-DENPADS are less than 10 FFU/well, which is comparable to the immuno-focus assay, except for with DENV-1 (Fig 5D). Considering the detection limit and time period of the assay, DENPADS is a sensitive and efficient way to titrate DENV.

The TCID50 method employs cell culture systems that reveal infectious virus through the development of virus-induced CPEs [7, 8]. The observation of the CPE is labor-intensive work with subjective variations between operators and laboratories. A serial dilution must be performed for each sample to obtain its endpoint titer. For plaque or immuno-focus assays, a serial dilution must also be made for each sample to acquire a number of plaques or foci within a countable range. Like observation of CPE, acquiring a sufficient number of plaques or foci requires intensive labor. Employing these methods to titrate samples is always a challenging and time-consuming task. In contrast, DENPADS can quantify virus samples between a titer <10 to >10^6^ FFU/well without any dilution (Figs 3A–3C and 5D). Importantly, acquiring data is easy when employing DENPADS. With these advantages, DENPADS allows more samples to be processes per operator.

Two classical methods, plaque assay and TCID50, are widely used to quantitate infectious virus. A monolayer of cells that is susceptible to virus infection with visible CPEs is necessary to support both methods. Some clinical strains or live attenuated virus (vaccine candidates) cannot be titrated by the two classical methods because CPEs do not occur after infection [9, 44]. Although development of a visible CPE is not required for immuno-focus assays, sophisticated staining processes and suitable antibodies are necessary. DENPADS does not have any of the above restrictions; it is easy to operate and can be used in a broad range of cell types.

High-throughput screening methods have been developed for discovering anti-DENV compounds in recent years. These methods include recombinant reporter DENV infection assays [45, 46], replicon-based assays [33, 34, 47–50], and a virus-like particle infection assay [51]. They are based on one model virus, which is selected at the beginning of system development. In contrast, DENPADS can be used to screen 4 DENV serotypes (Figs 2B, 5D and 5E). These results suggest that DENPADS can be applied to a broader range of DENV. Moreover, with those assays, it is difficult to distinguish whether the inhibition effect of anti-DENV drugs is a result of their specific ability to target and block virus replication or merely through a cytotoxic effect on normal cellular function leading to decreased production of replicon or reporter virus in cells. Two anti-DENV candidates, 2-CM and MPA, were used in this study. The results obtained from virus yield reduction assays and H-DENPADS were consistent with each other (Fig 6A–6D). Treatment with each drug candidate appeared to have inhibitory effects under DENV-2 infection. However, in the non-infection group, the OD45 nm values were invariant under 2-CM treatment but reduced in a dose-dependent manner under MPA treatment. This difference between 2-CM and MPA may be due to the slight cytotoxic effect of the candidates (Fig 6E and 6F). Cytotoxic effects restrict the cell numbers and lead to decreased OD450 nm values because the background OD450 nm values are associated with the total number of cells (S3C Fig). In line with these findings, the EC50 of 2-CM is dramatically lower than that of MPA [38, 39, 52]. These results suggest that H-DENPADS is an efficient method that can simultaneously evaluate cytotoxicity and potency of anti-DENV candidates when screening anti-DENV compounds.

In sum, DENPADS is a sensitive (low detection limits), fast (short time to complete the assay), accessible (easy to operate and low labor input), and high-throughput method.

## Supporting information

S1 Fig Localization of NS4B with or without present of NS2B3. BHK-21 cells were transfected with pSen-Cre. 24 hours post-transfect, localization of NS4B (red) and ER membranes (calnexin, green) were detected by immunostaining and nuclei were counterstained by Hoechst (magnification, 100×). The fluorescence intensity was analyzed by LEICA Application Suite software.
(PDF)

S2 Fig The detection of flavivirus protease. F-DENPADS cells were infected with Zika virus, and JEV at an m.o.i. of 0.05 to 5 or transfected with pHCV-Flag-NS3/4A. After 48hr, the presentation of flavivirus protease for each virus was detected by immunostaining using the anti-flavivirus NS3 (for Zika virus and JEV NS3) or anti-Flag (for HCV Flag-NS3/4A) primary antibody and HRP-conjugated secondary antibody. The NS3 protease presented by visible blue color after TMB membrane peroxidase substrates adding.
(PDF)

S3 Fig The virus yield reduction assays and the cytotoxic effect assay to anti-DENV compounds. Cells were exposed to DENV2 at m.o.i. 5 and incubated with increasing concentrations of two commonly used anti-flavivirus drugs, 2’-C-methyladenosine (2-CM) and Mycophenolic Acid (MPA). 24 hours later, (A)(B) The cytotoxic effect was analyzed by calculate the total numbers per well. (C) The background OD450 nm values were affected by the number of total cells. Different numbers of H-Den-ATCR cells were seeded into each well in 96-wells plate. After 24 hours incubation, the TMB substrate was added to react and the OD450nm value was measured.
(PDF)

S4 Fig The alignment of flavivirus amino acid sequences around the NS4B-NS5. (A) The sequence of the natural cleavage sites of the NS3 protease in the NS4B and NS4B/NS5 boundary of the polyprotein precursor. Identical residues are shaded in black and conserved residues are shaded in light grey. (B) Percent identity for flavivirus NS3 protease domain. The amino acid sequences of NS3 protease domain for DENV-1/Hawaii, DENV-3/H-87, DENV-4/H241, Zika virus/PRVABC59, JEV/Beijing-1 are aligned with DENV-2/16681 using the Vector NTI–AlignX software.
(PDF)

S1 Table Oligonucleotide primers used for PCR amplification. (PDF)

S1 Appendix STARD checklist. (PDF)

S2 Appendix STARD flowchart. (PDF)
