# Supplementary material for: Detection and quantification of dengue virus using a novel biosensor system based on dengue NS3 protease activity
Source: PLoS One. 2017 Nov 21;12(11):e0188170. doi: 10.1371/journal.pone.0188170 (PMC5697845; doi:10.1371/journal.pone.0188170)

S1 Fig. Localization of NS4B with or without present of NS2B3. BHK-21 cells were transfected with pSen-Cre. 24 hours post-transfect, localization of NS4B (red) and ER membranes (calnexin, green) were detected by immunostaining and nuclei were counterstained by Hoechst (magnification, 100×). The fluorescence intensity was analyzed by LEICA Application Suite software.

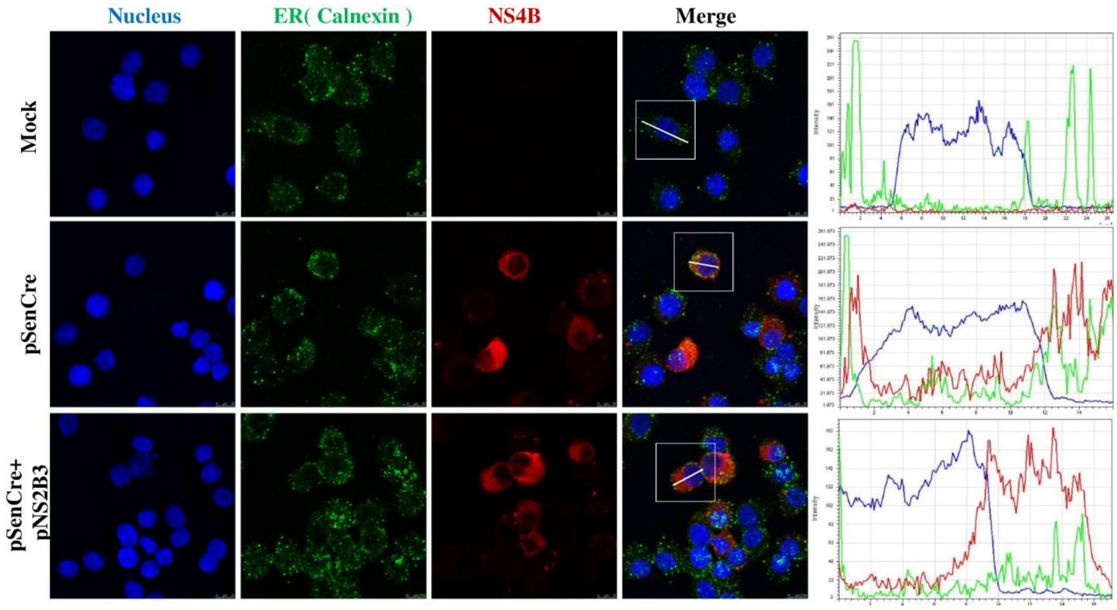

Supplement: S1 Fig — BHK-21 cells were transfected with pSen-Cre. 24 hours post-transfect, localization of NS4B (red) and ER membranes (calnexin, green) were detected by immunostaining and nuclei were counterstained by Hoechst (magnification, 100×). The fluorescence intensity was analyzed by LEICA Application Suite software. (PDF) [file pone.0188170.s001.pdf]
