# Supplementary material for: Detection and quantification of dengue virus using a novel biosensor system based on dengue NS3 protease activity
Source: PLoS One. 2017 Nov 21;12(11):e0188170. doi: 10.1371/journal.pone.0188170 (PMC5697845; doi:10.1371/journal.pone.0188170)
Supplement: S2 Fig — F-DENPADS cells were infected with Zika virus, and JEV at an m.o.i. of 0.05 to 5 or transfected with pHCV-Flag-NS3/4A. After 48hr, the presentation of flavivirus protease for each virus was detected by immunostaining using the anti-flavivirus NS3 (for Zika virus and JEV NS3) or anti-Flag (for HCV Flag-NS3/4A) primary antibody and HRP-conjugated secondary antibody. The NS3 protease presented by visible blue color after TMB membrane peroxidase substrates adding. (PDF) [file pone.0188170.s002.pdf]

S2 Fig. The detection of flavivirus protease. F-DENPADS cells were infected with Zika virus, and JEV at an m.o.i. of 0.05 to 5 or transfected with pHCV-Flag-NS3/4A. After 48hr, the presentation of flavivirus protease for each virus was detected by immunostaining using the anti-flavivirus NS3 (for Zika virus and JEV NS3) or anti-Flag (for HCV Flag-NS3/4A) primary antibody and HRP-conjugated secondary antibody. The NS3 protease presented by visible blue color after TMB membrane peroxidase substrates adding.

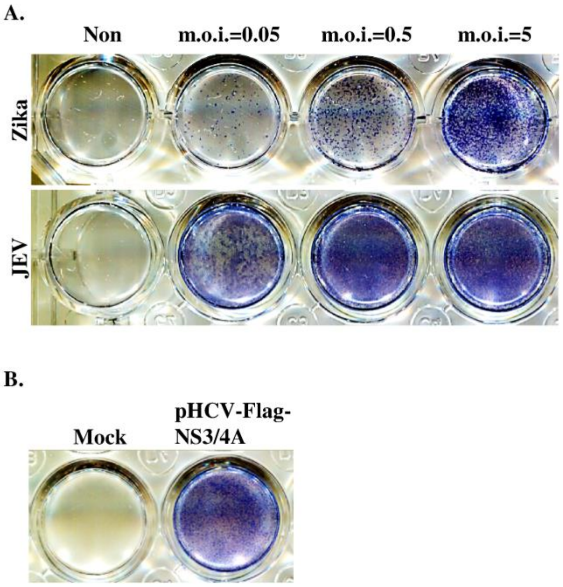

Materials and methods

Detection of flavivirus NS3 protease

F-DENPADS cells were seeding before infection at a density of 7 x 10^4 cells per well in 24-well plates. After 24 hours incubation, cells were infected by JEV or Zika or transfected with pHCV-Flag-NS3/4A. The infection or transfection protocols as described above. Cells were collected at 48 hours post-infection or post-transfection and fixed with IC Fixation Buffer (eBioscience) for 10 min. The cells were washed one time and permeabilizate with pereabilization buffer (eBioscience). Cells were then blocked with 3%BSA in PBS for 1 hours following incubated 1 hours at room temperature with primary anti-Flavivirus NS3 antibody (Yao-Hong Biotechnology) and or anti-Flag (GeneTex). After primary antibodies incubation following three times wash with PBS, the HRP-conjugated secondary antibody (Thermo Scientific) was added for 1 hour at room temperature following three times wash with PBS. The 3,3',5,5'-tetramethylbenzidine (TMB) membrane peroxidasesubstrates (Kirkegaard & Perry Laboratories, KPL, Gaithersburg, MD, USA) was added to each well and incubated for 10 min at room temperature. The flavivirus NS3 protease for each virus was detected by visible blue color.
