# Supplementary material for: Detection and quantification of dengue virus using a novel biosensor system based on dengue NS3 protease activity
Source: PLoS One. 2017 Nov 21;12(11):e0188170. doi: 10.1371/journal.pone.0188170 (PMC5697845; doi:10.1371/journal.pone.0188170)

S3 Fig. The virus yield reduction assays and the cytotoxic effect assay to anti-DENV compounds. Cells were exposed to DENV2 at m.o.i. 5 and incubated with increasing concentrations of two commonly used anti-flavivirus drugs, 2'-C-methyladenosine (2-CM) and Mycophenolic Acid (MPA). 24 hours later, (A)(B) The cytotoxic effect was analyzed by calculate the total numbers per well. (C) The background OD450 nm values were affected by the number of total cells. Different numbers of H-Den-ATCR cells were seeded into each well in 96-wells plate. After 24 hours incubation, the TMB substrate was added to react and the OD450nm value was measured.

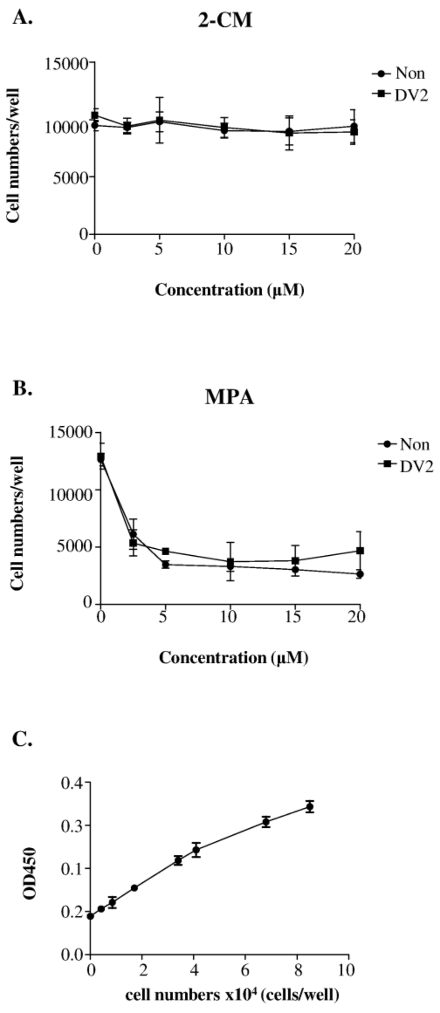

Supplement: S3 Fig — Cells were exposed to DENV2 at m.o.i. 5 and incubated with increasing concentrations of two commonly used anti-flavivirus drugs, 2’-C-methyladenosine (2-CM) and Mycophenolic Acid (MPA). 24 hours later, (A)(B) The cytotoxic effect was analyzed by calculate the total numbers per well. (C) The background OD450 nm values were affected by the number of total cells. Different numbers of H-Den-ATCR cells were seeded into each well in 96-wells plate. After 24 hours incubation, the TMB substrate was added to react and the OD450nm value was measured. (PDF) [file pone.0188170.s003.pdf]
