# Supplementary material for: Detection and quantification of dengue virus using a novel biosensor system based on dengue NS3 protease activity
Source: PLoS One. 2017 Nov 21;12(11):e0188170. doi: 10.1371/journal.pone.0188170 (PMC5697845; doi:10.1371/journal.pone.0188170)
Supplement: S4 Fig — (A) The sequence of the natural cleavage sites of the NS3 protease in the NS4B and NS4B/NS5 boundary of the polyprotein precursor. Identical residues are shaded in black and conserved residues are shaded in light grey. (B) Percent identity for flavivirus NS3 protease domain. The amino acid sequences of NS3 protease domain for DENV-1/Hawaii, DENV-3/H-87, DENV-4/H241, Zika virus/PRVABC59, JEV/Beijing-1 are aligned with DENV-2/16681 using the Vector NTI–AlignX software. (PDF) [file pone.0188170.s004.pdf]

S4 Fig. The alignment of flavivirus amino acid sequences around the NS4B-NS5. (A) The sequence of the natural cleavage sites of the NS3 protease in the NS4B and NS4B/NS5 boundary of the polyprotein precursor. Identical residues are shaded in black and conserved residues are shaded in light grey. (B) Percent identity for flavivirus NS3 protease domain. The amino acid sequences of NS3 protease domain for DENV-1/Hawaii, DENV-3/H-87, DENV-4/H241, Zika virus/PRVABC59, JEV/Beijing-1 are aligned with DENV-2/16681 using the Vector NTI–AlignX software.

A.

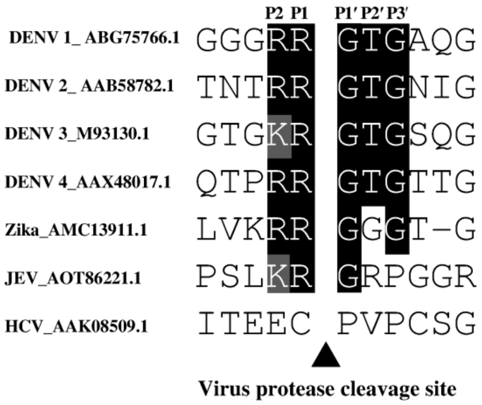

B.

	DENV 2	DENV 1	DENV 3	DENV 4	Zika	JEV	HCV
DENV 2	100	74	72	70	58	54	15
DENV 1		100	79	71	58	61	16
DENV 3			100	73	59	56	16
DENV 4				100	60	58	15
Zika					100	62	16
JEV						100	15
HCV							100
