# Supplementary material for: Detection and quantification of dengue virus using a novel biosensor system based on dengue NS3 protease activity
Source: PLoS One. 2017 Nov 21;12(11):e0188170. doi: 10.1371/journal.pone.0188170 (PMC5697845; doi:10.1371/journal.pone.0188170)
Supplement: S1 Table — (PDF) [file pone.0188170.s005.pdf]

S1 Table Oligonucleotide primers used for PCR amplification.

Oligonucleotide	Sequence[a]
XhoI-Kozak-NS4B-F	5'-GGGCCCTCTAGA*CTCGAG***GCCACCATG**GCAAACGAGATGGGTTTCCTAGAAAAAACG-3'
AflII-EcoRI-(N_{10}-NS5)-R	5'-GTTTAAAC*TTAAGC*C*GAATTC*CTCTCCAAGCGTCTCTCCTATG-3'
EcoRI-nCre-F	5'-ATCTGCA*GAATTC*CCCAAGAAGAAGAGGAAGGTGTCCAATTTACTGACCGTACA-3'
XhoI-*NotI*-Cre-F	5'- TCTAGA*CTCGAGCGGCCGC*ATGTCCAATTTACTGACCGTAC -3'
AflII-*HindIII*-Cre-R	5'-GTTTAAAC*TTAAGCTT*CTAATCGCCATCTTCCAGCAGGCGC-3'
XhoI-*NotI*-NS2B-F	5'-TCTAGA*CTCGAGCGGCCGC*ATGAGCTGGCCATTAAATGAGGCTA-3'
AflII-*HindIII*-NS3-R	5'-CGGCAC*TTAAGCTT*CTACTTTCTTCCGGCTGCAAATTCC-3'
BamHI-EGFP-F	5'-AATTAA*GGATCC*ATGGTGAGCAAGGGCGAGGAG-3'
XbaI-EGFP-R	5'-CGTGCC*TCTAGA*TTACTTGTACAGCTCGTCCAT-3'

[a] italics, the restriction endonuclease; bold text, the Kozak sequence; underlined text, SV40 NLS.
