# Supplementary figures and images for: Detection and quantification of dengue virus using a novel biosensor system based on dengue NS3 protease activity
Source: PLoS One. 2017 Nov 21;12(11):e0188170. doi: 10.1371/journal.pone.0188170 (PMC5697845; doi:10.1371/journal.pone.0188170)

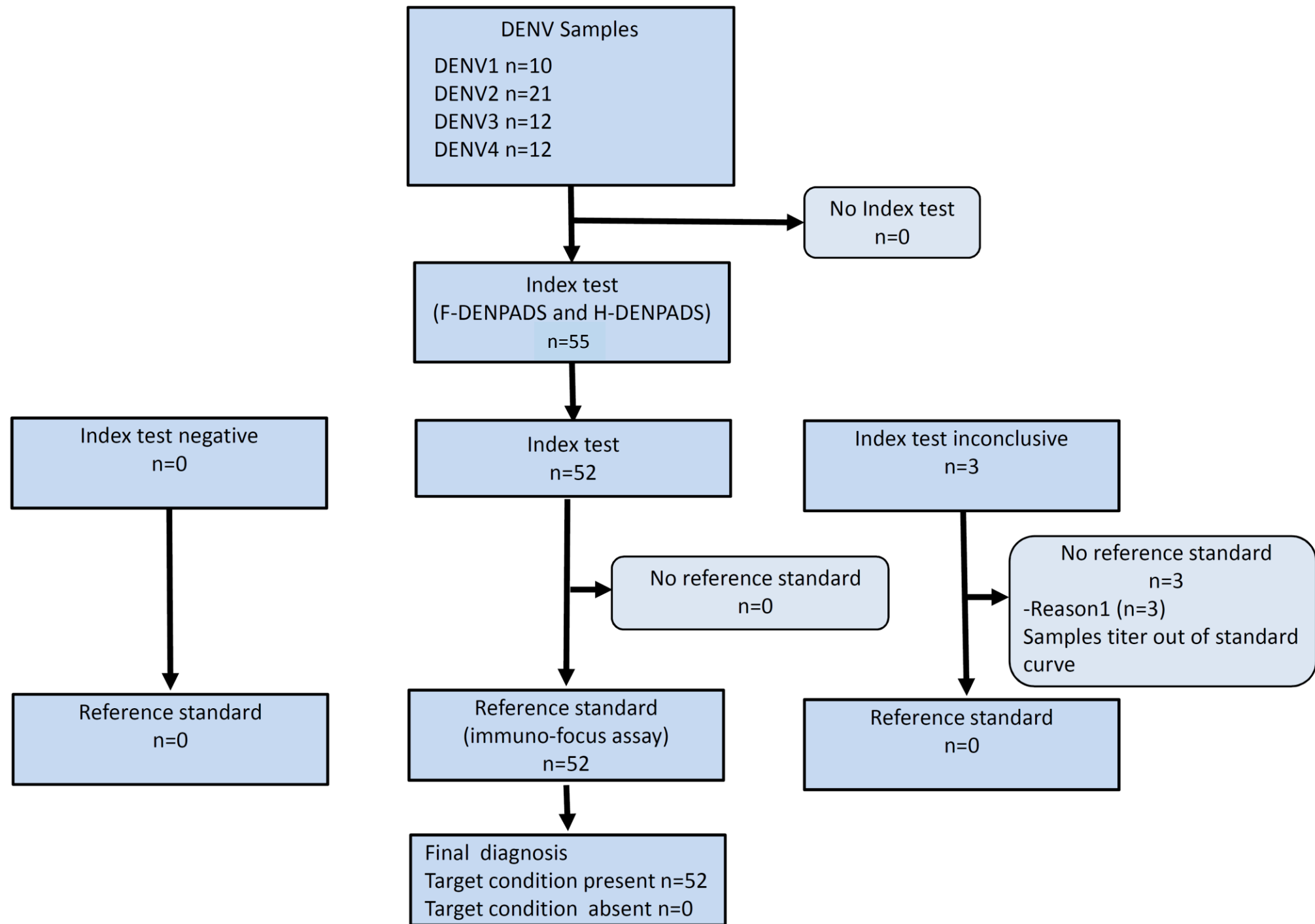

Supplement: S2 Appendix — (PDF) [file pone.0188170.s007.pdf]
